# Sensor-Based Diagnostics for Conveyor Belt Condition Monitoring and Predictive Refurbishment

**DOI:** 10.3390/s25113459

**Published:** 2025-05-30

**Authors:** Ryszard Błażej, Leszek Jurdziak, Aleksandra Rzeszowska

**Affiliations:** Faculty of Geoengineering, Mining and Geology, Wroclaw University of Science and Technology, Na Grobli 15 St., 50-421 Wrocław, Poland; ryszard.blazej@pwr.edu.pl (R.B.); leszek.jurdziak@pwr.edu.pl (L.J.)

**Keywords:** conveyor belt, refurbishment, predicting maintenance, condition monitoring, circular economy

## Abstract

Rising raw material costs and complex global supply chains have reduced the durability and availability of conveyor belts. In response, condition-based maintenance (CBM) with in situ diagnostics has become essential. This case study from a Polish lignite mine shows how subjective visual inspections were replaced with objective, repeatable measurements of belt core condition and thickness. Shifting refurbishment decisions from the plant to the conveyor improved success rates from 70% to over 90% and optimized belt lifecycle management. Sensor-based monitoring enables predictive maintenance, reduces premature or delayed replacements, increases belt reuse, lowers costs, and supports the circular economy by extending belt core life and reducing raw material demand. The study demonstrates how real-time, sensor-based diagnostics using inductive and ultrasonic technologies supports predictive maintenance of conveyor belts, improving refurbishment efficiency and lifecycle management.

## 1. Introduction—Global Conveyor Belt Market

Belt conveyors are mechanical systems consisting of continuous belt loops used for transporting bulk or unit materials over short to long distances, including within buildings, storage yards, assembly lines, and supply chains—sometimes across hundreds of kilometers [1,2].

The transported material rests on the belt’s top cover and is driven by pulling forces within the core. The system typically consists of at least two pulleys, one of which drives the loop. Due to the low belt-to-material mass ratio, conveyor belts are among the most cost-effective transportation systems [3]. Their versatility makes them widely used in mining [4], cement manufacturing [5], power generation [6,7], recycling [8], metal processing, chemicals [9], oils, gases, food and beverage processing [10], general manufacturing, supply chain, automated distribution, warehousing [11], airports [12], and other sectors. Increasing deployment of belt systems contributes to reducing production costs, supporting overall market growth.

This growth is further fueled by technological advances, rising demand for automation, and the need for condition monitoring, diagnostics, and environmental sustainability. Electric-powered conveyors are also considered more eco-friendly than fossil-fuel alternatives. Despite market expansion, challenges remain—especially for small and medium enterprises—due to high belt procurement, installation, and maintenance costs, exacerbated by rising electricity prices, particularly in the European Union. Solutions such as belt refurbishment [13,14,15], energy-saving belts [16,17] and structures [18,19,20] and intelligent management strategies [6,21] are gaining importance.

The market includes textile-reinforced belts, steel cord belts, and solid woven types [22,23]. Textile belts (EE and EP) are projected to remain dominant due to their cost-effectiveness. Steel cord belts are favored in mining for their superior impact resistance and strength [24,25,26], though rising steel prices affect market value.

While growing demand is expected in developing countries, the shift away from coal-fired power in industrialized nations presents a challenge. Conveyor belts, especially heavy-duty ones, require large quantities of raw materials, which account for 70–75% of total production costs and strongly influence product quality [27].

**Figure 1:**
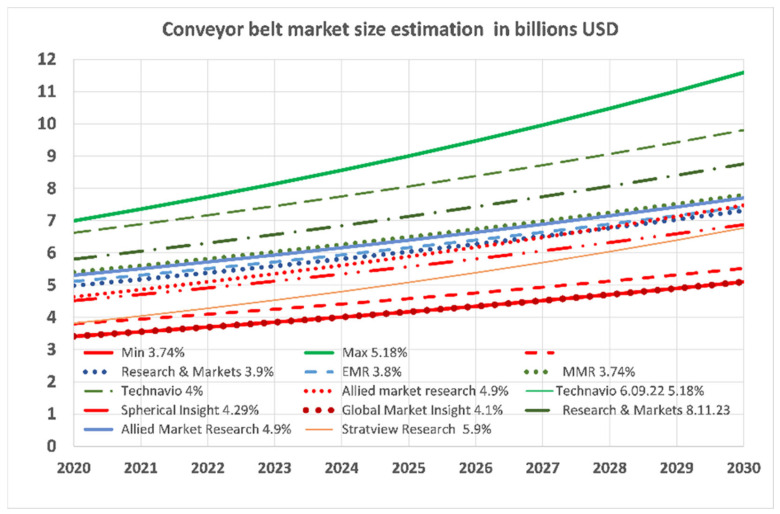
Global conveyor belt market size and forecasted growth measured in billion dollars. The Compound Annual Growth Rate (CAGR) of the belt market growth between 2023 and 2032 are given along with the names of market research firms and the date of report publication.

Market size estimates vary widely, ranging from over $3 billion in 2020 to $11.5 billion by 2030, with projected CAGR between 3.74% and 5.9%. These discrepancies stem from differing methodologies and segmentation criteria (e.g., belt types, construction materials, applications, and geography), as illustrated in Figure 1.

As belts are the costliest component of conveyor systems, their condition must be closely monitored. Predictive diagnostics and preventive replacement strategies not only reduce downtime but also promote material reuse and sustainability.

The pandemic, wars and the imposition of customs barriers disrupt the global supply of raw materials, causing material difficulties, extending the routes and delivery times of components for production and finished products. It also affects the increase in the prices of belts and lowers their durability when good quality components are replaced with cheaper substitutes.

In a situation of supply uncertainty, the strategy of extending the life of products through their refurbishment becomes a rational choice. It leads to cost and rare raw materials savings. The core of St type belts can be used three times, because in Poland, conveyor belts in brown coal mines are refurbished twice. In the case of the Bełchatów lignite mines, when one of two pits is being liquidated due to the lignite depletion, the mine has a large number of used belts. Their use by transferring them to other conveyors or refurbishment deepens the rationality of refurbishment, as it almost completely eliminates the need to purchase new belts. Considering that the cost of recondition is half the cost of purchasing a new belt, it is also economically justified. This study evaluates the cost-effectiveness of preventive belt replacement strategies integrated with refurbishment practices. The proposed methodology has been successfully applied in Polish lignite mines for over five decades and aims to optimize belt lifecycle management through condition-based diagnostics. Unlike conventional approaches relying on calendar age and visual cues, the proposed system uses objective, in situ diagnostics to evaluate the internal condition of steel-cord belts in real time, allowing optimized refurbishment decisions.

The innovation of this study lies in applying in situ magnetic diagnostics to quantify internal core damage in steel-cord belts, replacing subjective visual inspections with objective, data-driven assessments that improve refurbishment success rates.

## 2. Strategy of Belt Replacements

The market value of conveyor belts includes both the installation of new belts on investment conveyors and the replacement of worn sections on existing ones. A belt loop on long conveyors consists of multiple spliced sections (vulcanized, adhesive, or mechanical). New loops are built from the longest possible sections to minimize the number of splices, which represent structural weak points. Belts are typically delivered on single (250–400 m) or double reels (up to 700+ m). During operation, damaged segments or joints are replaced, gradually shortening section lengths and increasing the number of splices over time [28]. This process is referred to as belt renewal [29,30,31], which may involve new, less worn, or refurbished belts.

Belt degradation is influenced by many factors: conveyor design, belt construction, material type, working conditions, and operational regime [32,33,34,35,36]. Because these factors interact dynamically over time, belt lifetime is difficult to predict and should be treated as a random variable. Attempts to model belt durability based on operational statistics remain limited due to complexity, subjective replacement decisions, and inadequate metrics (e.g., calendar time instead of effective operational hours or transported mass) [37,38,39,40]. As a result, belt durability varies significantly, and its evaluation requires multicriteria analysis [41,42,43,44].

Furthermore, unit costs of belts with identical specifications may vary twofold. A lower price typically reflects lower material quality, but a high price does not guarantee longer lifespan. Without predictive tools, durability cannot be reliably assessed in advance.

While data on the new belt market is available, the refurbished belt market remains poorly quantified. Refurbishment was once common and widely offered by major service providers. Today, only selected suppliers (e.g., Fenner Dunlop Conveyor Belting (Pittsburgh, PA, USA), ContiTech AG (Hanover, Germany)) and service firms (e.g., Bestgum Sp. z o.o. (Rogowiec, Poland)) continue this service. Most users opt for new belts and consider refurbishment only after early-stage damage. Only a few, such as Polish lignite mines, apply refurbishment as a regular replacement strategy.

Failures caused by external stochastic events (e.g., punctures, cuts) may occur regardless of belt age or condition. The likelihood increases as belts wear, but accurate prediction would require extensive failure case data, which many operations do not collect or publish.

Ultimately, the decision to replace, repair, or refurbish depends on economic trade-offs. If repair downtime is shorter than replacement time, in situ repairs are preferred. Otherwise, belts are dismantled and refurbished either on-site or in specialized plants.

**Figure 2:**
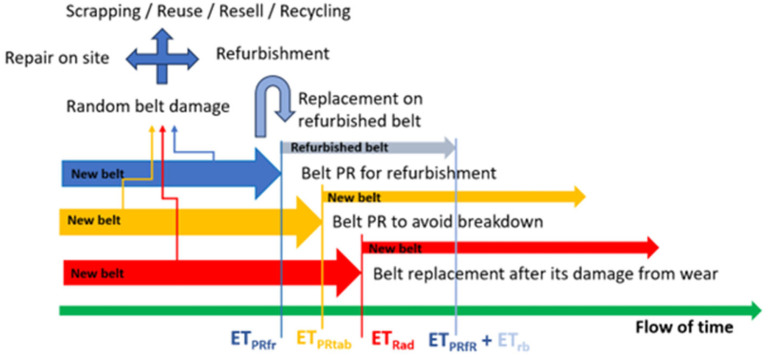
Expected times (ET) of belt replacements in different replacement strategies.

Figure 2 shows the expected runtimes for different replacement policies. The earliest belts to be replaced are those replaced preventively for refurbishment. The point is to ensure that overuse of the belt does not cause damage to the belt core precludes the belt from being refurbished with success. Corrosion of the cables in the core in many places with numerous punctures will reduce the adhesion of the cables to the rubber, and the need to make many repairs may increase the costs and time of refurbishment. A reconditioned belt may not pass the tests allowing it to be used, or the refurbishment costs will be too high compared to purchasing new belts.

The belt segment can be damaged randomly at any age. In such cases, the maintenance supervisor has to decide what to do with failures. They can be repaired on site (at once, at the closest planned standstill, and later when more small failures occur). When failures require a long time to be repaired on-site and create a threat to continuous operation, it is better to replace the belt fragment or the whole segment on a new one and send the belt for refurbishment. If the belt is not worth repairing, it can be scrapped, or reused for another purpose (e.g., as seals, shock-absorbing linings, fenders, construction mats, marine dock bumpers, truck bed liners, grain pit covers, etc.). They can be resold to less demanding users or recycled. The last solution requires a special line for worn-out belt cover milling, cutting the core into smaller parts, scrubbing, separating, etc. Such lines will be required to utilize a lot of old belts when coal mines are closed shortly.

Decisions regarding sending the belt for refurbishment must be economically justified. If the cost of refurbishment the belt is $C_{rb}$, and the cost of purchasing a new belt is $C_{nb}$, then the unit cost of using a new belt will be the quotient $\frac{C_{nb}}{ET_{PRtab}}$ (where *ET_PRtab_* is expected time in preventive replacement to avoid breakdown strategy), and for the refurbished belt, it will be $\frac{C_{rb}}{ET_{PRfr}}$ (where *ET_PRfr_* is expected time in preventive replacement for refurbishment). Refurbishment is cost-effective if both conditions (1) and (2) are met.

(1) $$\frac{C_{rb}}{ET_{PRfr}}<\frac{C_{nb}}{ET_{PRtab}}$$

(2) $$C_{rb}<C_{nb}\cdot \frac{ET_{PRfr}}{ET_{PRtab}}$$

If the cost of refurbishment is 50% of the price of a new belt, then even when their lifespan is shorter than that of new belts but exceeds 50%, the use of refurbishment is cost-effective. Because not all belts sent for refurbishment can be successfully reconditioned, the relationship should be modified by introducing a refurbishment success rate indicator, showing the percentage of belts sent for refurbishment that are successfully reconditioned (RSR—Refurbishment Success Rate). It can be assumed that this rate depends on the time of belt usage and changes from 1 to 0.

To determine the unit cost of using 1 m of refurbished belts when RSR(t) is less than 1, more belts need to be sent for refurbishment because some may not be successfully refurbished. The length of belts sent for reconditioning is obtained by dividing 1 by RSR(t). Over time, as RSR(t) decreases, the length of belts sent for refurbishment will increase according to the function $\frac{1}{RSR\left(t\right)}$ (Equations (3) and (4)).

(3) $$\frac{C_{rb}}{RSR\left(t\right)\cdot ET_{PRfr}}<\frac{C_{nb}}{ET_{PRtab}}$$

(4) $$C_{rb}<C_{nb}\cdot RSR\left(t\right)\cdot \frac{ET_{PRfr}}{ET_{PRtab}}$$

If the $RSR\left(t\right)$ indicator at the time of replacement is 0.7, and the ratio of the durability of refurbishment and new belts is, for example, 80%, the inequality will be true since 50% of $C_{rb}<C_{nb}\cdot 0.7\cdot 80\%$ and $C_{rb}<C_{nb}\cdot 56\%$. Knowing the course of the $RSR\left(t\right)$ function, you can determine the critical time, $T_{rbt}$, from Equations (5) and (6).

(5) $$C_{rb}=C_{nb}\cdot RSR\left(T_{rbt}\right)\cdot \frac{T_{rbt}}{ET_{PRtab}}\;$$

(6) $$T_{rbt}\cdot RSR\left(T_{rbt}\right)=ET_{PRtab}\cdot \frac{C_{rb}}{C_{nb}}\;$$

If all belts could be successfully refurbished and incorporated into production, $RSR\left(t\right)$ would be equal to 1 (at least up to $T_{rbt}$), and then (7).

(7) $$T_{rbt}=ET_{PRtab}\cdot \frac{C_{rb}}{C_{nb}}$$

If, thanks to the diagnostics of the belt’s condition, it were possible to determine the belt wear index $I_{bc}(t)$ and its changes over time, as well as the critical state $I_{bc}\left(t\right)=I_{cr}$, after reaching which the belt should be dismantled and sent for refurbishment, then the replacement moment would be chosen not based on time, which is not a good measure of belt wear, but based on its condition, determined by reliable and objective methods. For this time, RSR(t) can be calculated and checked to see if it ensures cost-effective refurbishment.

**Figure 3:**
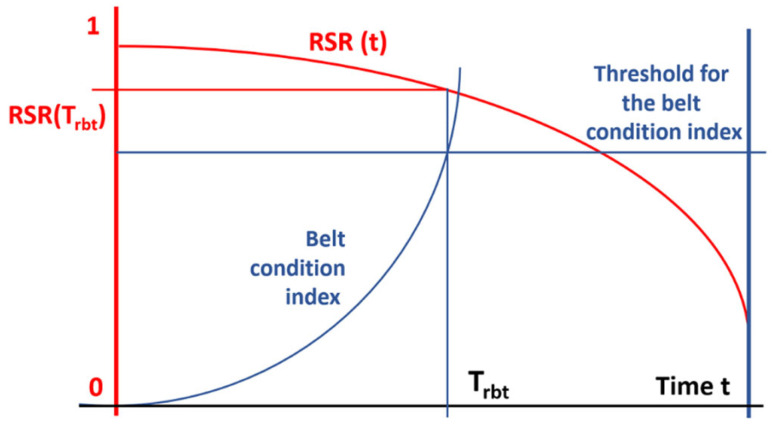
Method of determining the replacement time $T_{rbt}$ based on the hypothetical deterioration of the belt condition $I_{bc}\left(t\right)$ until reaching the critical value. The intersection point determines the optimal replacement time, and this allows the calculation of $RSR(T_{rbt})$ to verify if cost-effectiveness is maintained.

Figure 3 illustrates the proposed method for determining the optimal replacement time based on projected failure density over time, which forms the basis of the predictive replacement algorithm.

The Belt Condition Index (I_bc_(t)) is a quantitative measure of belt wear. At the Bełchatów lignite mine, a related metric—failure density—is commonly used. This is defined as the number of detected failures on a given belt segment divided by its length. This approach allows for meaningful comparisons of wear across belts of varying lengths and ages.

Failure density can serve multiple purposes. Locally, it helps identify specific sections of the belt that may require targeted repair or replacement. On a broader scale, it can be applied to entire belt sections to determine the optimal timing for removal and refurbishment.

Threshold values for I_bc_ and failure density are selected empirically by the mine’s belt maintenance team and refurbishment plant. These teams analyze the Refurbishment Success Rate (RSR) for belts with different I_bc_ values to guide threshold selection.

The critical time for preventive replacement—denoted as T_rbf_—is established at the point where the I_bc_(t) trend line intersects the defined threshold (see Figure 3). Validation of this threshold is also empirical: if belts with lower failure densities fail refurbishment criteria (e.g., strength tests), the threshold is adjusted downward. Conversely, if all belts at the threshold density pass refurbishment successfully, the threshold may be slightly increased.

In essence, RSR should be determined not for time but for the condition of the belt.

## 3. The Beginning of Conveyor Belt Refurbishment in Poland [45]

**Figure 4:**
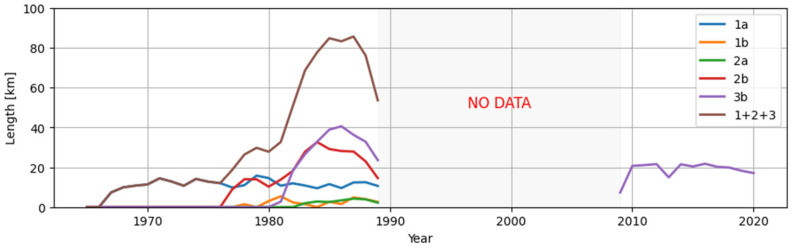
Reconditioning of conveyor belts at the Polish lignite surface mines: Turow—1, Konin—2, Belchatow—3. a—textile belt, b—steel cord belts. Data from [45].

Since the mid-1960s, conveyor belts have been reconditioned in lignite mines in Poland. The first reconditioning department was set up in the Turow lignite surface mine (1a, 1b, Figure 4) The next was started up in 1977 at the Konin mine (2a, 2b, Figure 4) and in 1981 reconditioning began at the biggest and most modern lignite mine Bełchatów (3b, Figure 4).

Although the data in this section originate mainly from the 1980s, they are included to illustrate the long-term scale and historical significance of conveyor belt refurbishment practices in Poland’s lignite industry. These insights provide a valuable context for understanding how current refurbishment strategies have evolved and how they compare to present-day practices supported by modern diagnostics.

In Figure 4, we can see that the maximal total length of reconditioned belts was close to 85 km in mid 80-ties, which gives about 14.8% of the total length (573 km) of installed belts. The lifespan of naturally worn-out belts is 49 months (4.1 years). Refurbished belts attain 85.7% of it (42.9 months = 3.6 years). With the refurbishment costs lower than 50% of a new belt price. We have profitable refurbishment, due to 85.7% is greater than 50%. Even for all belts removed the durability of refurbished belts was greater than 50% (min. 54.3%; max. 62.9%). The quality of refurbished belts has increased since the mid-80-ties. One of the reasons is that this time, the belt management computer system “Tasma” has been applied in the Belchatow mine which helped in rational belt management and maintenance decisions. The success rate of belt recondition can be as low as 58.34% due to 50% = 87.5% · 58.34%. This provies a good margin for wrong decisions about belt replacements. Data published in the paper [45] allows for the estimation of the proportion of new and reconditioned belts to all belts supplied the mine between 1981 and 1989 to be 58.65% and 41.35%, respectively.

**Figure 5:**
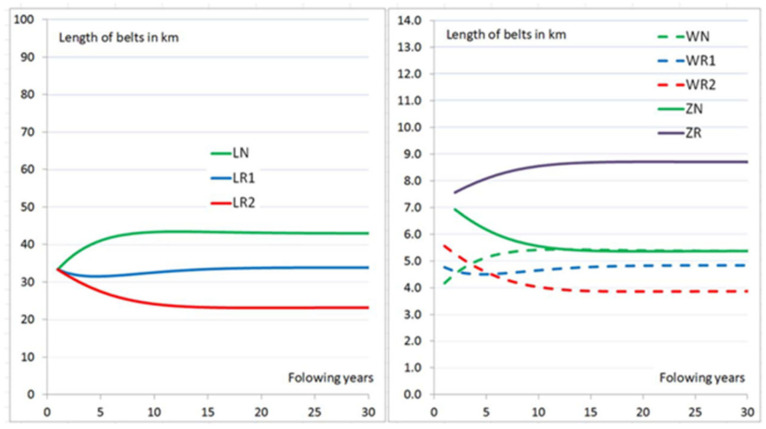
Stabilization process of belt length installed in the mine (**left**) and demanded (**right**) in the simulated strategy (deterministic variant) of belt replacements for recondition at the hypothetical mines having 100 km belts installed. Installed belts: new LN, after 1st reconditioning LR1, after 2nd reconditioning LR2), replaced belts (WN, WR1 and WR2), the newly purchased ones (ZN). Data from [46].

A similar proportion of new belts, belts after the 1st recondition and the 2nd recondition, estimated for the conditions of one of our lignite surface mines, are a bit different. For a hypothetical mine with 100 km belts installed, proportion stabilizes, as is shown in Figure 5.

**Figure 6:**
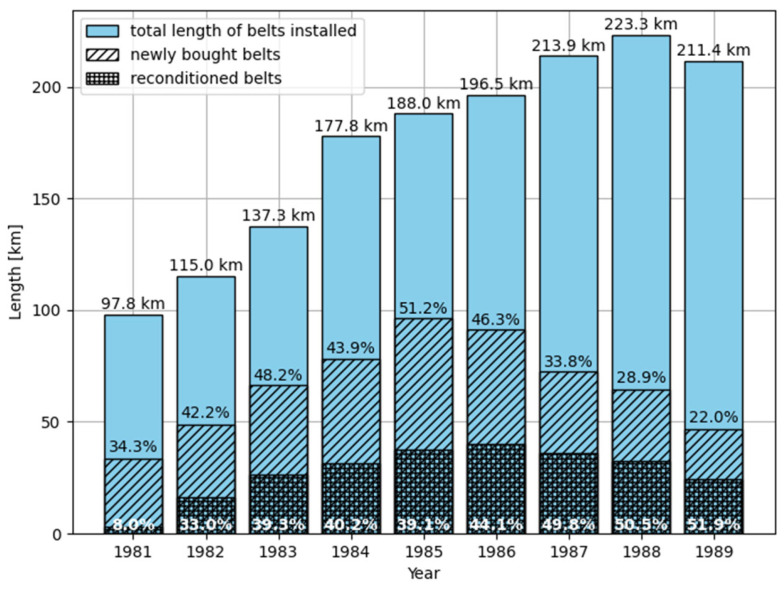
The ‘Belchatow’ open pit mine demand for belts concerning the length of belts installed. Data from [45].

The initial state for iterative calculations of belt replacements including high refurbishment success rate RSR=0.9 and $RSR_{2}=0.8$ (in reality, it is about 0.7). All types of belts new (LN), after the 1st recondition (LR1) and after the 2nd recondition (LR2) have the same length of 33.3 km. After several years of belt replacements, as is seen with new belts that are 43 km in length and refurbished belts 57 km in length. Among the replaced belts, refurbished made up 58.65% and new ones 41.35%. Real data in the past were the opposite. The new belts supplied to the mine for 10 years made up 58.65% and refurbished ones made up 41.35% (Figure 6). The differences came from the fact that demand for belts was not so stable due to changes in the length of conveyors’ routes (mainly their increase from 50 km up to 112 km due to pit development—the length of belts is twice as much). The durability of reconditioned belts was much lower than that of new belts (slightly over 60% for the whole period and 85.7% in 1989) and unknown RSR which was much lower than the current value, especially after the application of diagnostics to select the best moment of belt dismantling for recondition.

## 4. Sensor-Based Diagnostic Systems for Predictive Belt Refurbishment

### 4.1. Overview of Diagnostic Straregy

Various techniques have been developed to assess the condition of conveyor belts, particularly those with steel cords. Among them, X-ray imaging systems are often cited for their high diagnostic accuracy in detecting internal structural damage. However, these systems are rarely used in Polish industrial practice due to several limitations: they are expensive to install and operate, involve radiation safety concerns, and require specialized staff to interpret radiographic images manually. Additionally, the need to halt operations and isolate belt sections for scanning significantly reduces their applicability for routine monitoring.

In contrast, the DiagBelt+ system provides a non-invasive, in situ diagnostic solution that scans the belt loop while in motion, without disassembly or interruption of conveyor operation. The system uses magnetic field distortion analysis to identify discontinuities in steel cords and areas of weakened adhesion. The results are automatically visualized and quantified, enabling objective assessment by technical staff and eliminating the subjectivity inherent in visual inspection or X-ray interpretation.

BeltSonic complements DiagBelt+ by assessing the condition of the rubber cover using ultrasonic measurements. This combination provides a comprehensive evaluation of both the core and surface condition, supporting more accurate decisions about refurbishment, continued use, or disposal. Together, these systems offer a practical, scalable alternative to existing technologies, adapted to real industrial constraints.

### 4.2. Sensor System Architecture

The diagnostic framework employed in this study integrates two complementary sensing technologies: the DiagBelt+ system, based on inductive magnetic field sensors for core integrity assessment, and the BeltSonic system, based on ultrasonic distance sensors for measuring cover thickness and belt sagging. Both systems are designed for in situ diagnostics of moving conveyor belts, eliminating the need for belt disassembly or operational downtime.

DiagBelt+ employs a set of inductive sensors arranged within a measuring head, which is mounted on a stationary frame surrounding the conveyor belt. The system also includes permanent magnet strips that magnetize the steel cords embedded in the belt’s core. As the belt moves, magnetic field disturbances caused by damage (e.g., cord breakage, corrosion, splice defects) are detected by the coils and converted into voltage signals.

The measurement resolution is typically one coil per 1–2 cords, allowing high spatial granularity. A tachometer mounted on the belt frame ensures synchronization of spatial measurements. The resulting magnetic signal is processed to produce a two-dimensional defect map of the entire belt loop. Parameters such as failure density (defects per meter), splice locations, and damage types are extracted using proprietary algorithms.

The BeltSonic module consists of two ultrasonic heads placed on opposing sides of the belt. Each head contains high-frequency transducers (typically 40–60 kHz), which measure the distance to the belt surface by analyzing the time-of-flight of reflected ultrasonic pulses. Knowing the fixed distance between the heads, the system computes the local thickness of the belt.

This differential measurement technique enables continuous scanning of belt cover thickness, sagging, and longitudinal/transverse deformations. Measurements can be repeated periodically, allowing trend analysis of belt wear over time. The system outputs a wear map of the belt, showing average and minimum cover thickness and percentage material loss relative to nominal specifications.

Both sensor systems are connected to local data acquisition (DAQ) modules, which digitize and preprocess the signals. Sensor data are transmitted to a central diagnostic computer. Custom software (DiagBelt+ Data Analysis v. 1.0) aggregates and visualizes the data, performing threshold analysis, condition indexing, and degradation trend forecasting.

**Figure 7:**
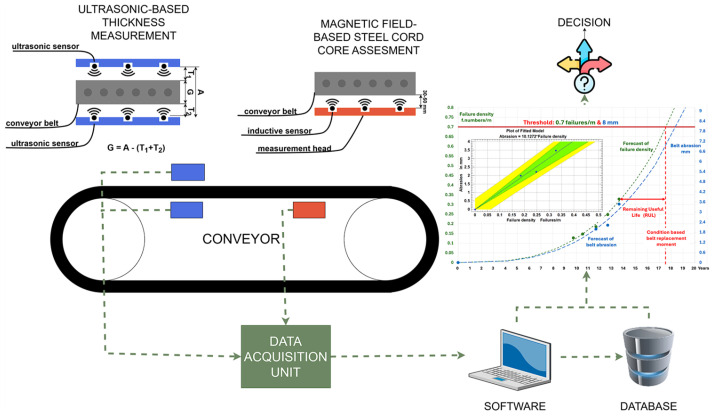
Overall architecture of the sensor-based conveyor belt diagnostic system.

A schematic of the integrated sensor system and data flow is presented in Figure 7. This diagram illustrates the physical arrangement of the sensors, data flow pathways, and analytical processing blocks that support decision-making.

The diagnostic outputs generated from sensor data—such as Belt Segment Condition Index (BSCindex), failure density profiles, and cover thickness loss—are used in a predictive maintenance algorithm. Each belt segment is evaluated against empirically defined thresholds to determine whether it is:

- fit for continued operation,
- eligible for refurbishment, or
- in need of scrapping or replacement.

This sensor-driven approach replaces subjective, visual assessments with quantitative diagnostics, significantly improving decision accuracy and enabling condition-based refurbishment planning. Results from full-scale deployment in the Bełchatów lignite mine confirm the practical value of this architecture: refurbishment success rates increased from 70% to over 90%, while belt utilization time was significantly extended in many cases.

### 4.3. Data Interpretation and Condition Indices

**Figure 8:**
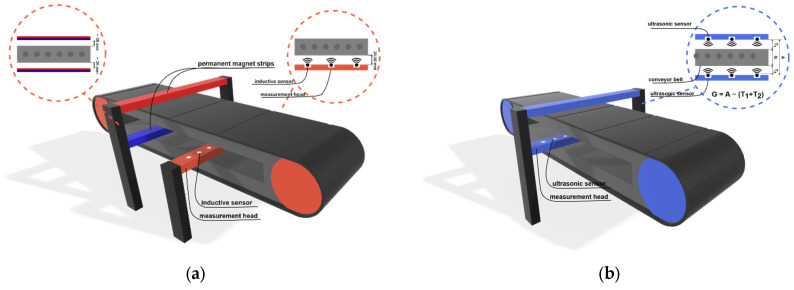
Schematic diagram of the measurement system installation on the conveyor belt. (**a**) DiagBelt+ system (**b**) BeltSonic system.

To accurately assess the technical condition of conveyor belts and make informed decisions regarding refurbishment at the right time, it is necessary to have information about both the condition of the belt’s core and the thickness of its covers [13]. The DiagBelt+ system, developed at the Wrocław University of Science and Technology, utilizes a magnetic method to assess the technical condition of the core with steel cords, while the BeltSonic system uses a differential measurement method to determine the thickness of the measured object. Both of these systems are installed around the moving conveyor belt, as shown in Figure 8. The conveyor belt core diagnostic system (DiagBelt+ [47]) requires the installation of permanent magnet strips, which are intended to magnetize the core links of the belt. It also includes a measuring head equipped with magnetic coils that record changes in the magnetic field at the location of discontinuities in the links. On the other hand, the thickness measurement system (BeltSonic [48]) has two measuring heads installed parallel on both sides of the conveyor belt. Ultrasonic sensors installed in them measure the distance to the supporting and running covers of the belt, and knowing the distance between the sensors, allows for a precise determination of the thickness of the belt at a given location.

The BeltSonic system is designed for measuring the thickness and assessing changes in the transverse and longitudinal profiles of various types of conveyor belts. The operating principle of this system is based on a differential measurement. Measurement heads, placed above and below the examined object, have ultrasonic sensors that measure the distance from the sensor to the obstacle, i.e., the cover of the conveyor belt. By knowing the distance between the measurement heads and readings from both sensors (located above and below the belt), the thickness of the examined object can be determined unambiguously. The operation of the system and its design parameters have been detailed in works [49].

### 4.4. Diagnostic Maps and Visualization

**Figure 9:**
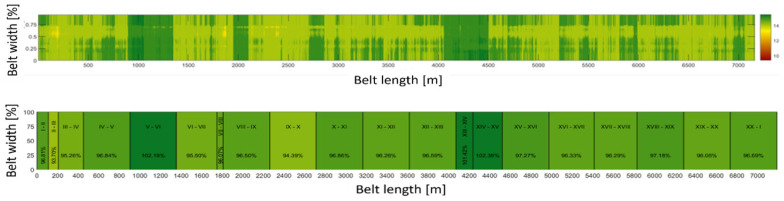
Conveyor belt loop map with sections—assessment of the percentage loss relative to the original thickness of the covers of the conveyor belt section—BeltSonic system.

Measurements using the BeltSonic system enable the determination of the average and minimum thickness of the examined belt section, as well as the calculation of the percentage loss of its surface. Conducting measurements at specified time intervals allows for monitoring the rate of belt wear [33,40]. Analyzing the results of measurements for individual sections (or shorter fragments of the conveyor belt) and comparing the measured thickness with the nominal thickness of the conveyor belt provides an image of the wear of individual sections of the examined object. An exemplary contour map of a performed measurement and a wear measure map (average percentage loss of the original cover thickness calculated for the entire surface of the section between connections) are presented in Figure 9 for an extended loop of a belt consisting of twenty sections.

The DiagBelt+ system is designed to detect and record changes in the magnetic field that occur at the belt splice or in areas of core damage. The most common failures include corrosion of the links, cuts in the links, or their absence on a specific section of the belt (e.g., a few centimeters long). It consists of a measurement head, which is equipped with coils to read changes in the magnetic field, two strips of permanent magnets to magnetize the core, a tachometer, and a data acquisition module. A detailed description of the system’s operation can be found in works [13,50].

**Figure 10:**
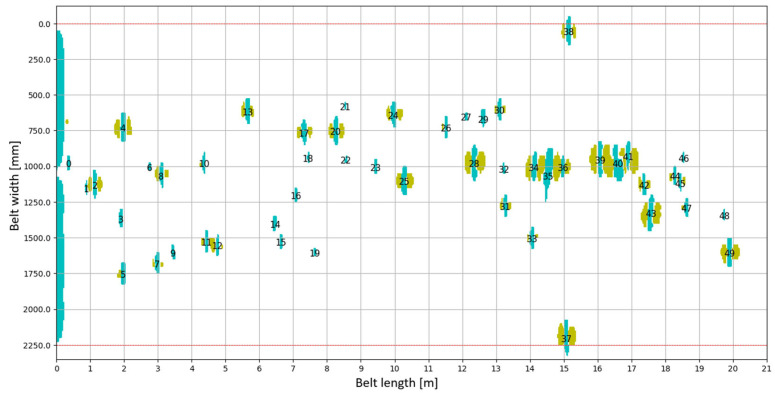
Visualization of measurement data—an example of a two-dimensional image from the DiagBelt+ system. Cyan indicates a signal value of +1, and yellow indicates a value of –1 (associated signal). The numbers mark consecutive identified belt failures.

Thanks to measurements taken by the DiagBelt+ system, it is possible to obtain an image of failures, which, in turn, allows assessing the degree of damage to the belt’s core and verifying the correctness of the joint’s execution and its technical condition. By analyzing the number of failures, their density on a given belt section, and the characteristics of failures (their shape and belonging to specific categories), it is possible to determine which section of the belt requires repair. Figure 10 shows signals recorded by the DiagBelt+ system, which are then used for damage analysis.

**Figure 11:**
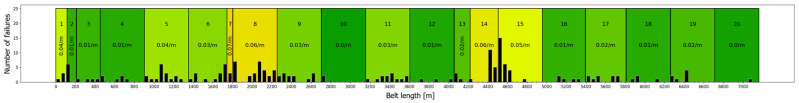
Map of aggregated wear measure (here, average density of failures per 1 m for the entire section of the belt) for the extended loop of the conveyor belt—assessment of the technical condition of the belt core—image from the DiagBelt+ system.

The diagnostic system also includes software that, based on measurement data, generates an assessment report of the technical condition of the examined object. This program takes into account various parameters and drawings, such as the number of failures, the quantity of splices, and the length of the belt. After analyzing the data, the system generates a map of the technical condition of the examined conveyor belt, covering all sections of the belt loop. On this map, by setting thresholds beforehand, it is possible to visually assess the condition of a particular section, as the failures represented on the map correspond to the density of failures. An example of such a map is presented in Figure 11.

Analysis of the distribution of registered failures on histograms, visible in the report, allows for determining which part of the belt is most susceptible to failures.

The DiagBelt+ system represents a shift from visual, experience-based judgments to quantitative magnetic measurements of core damage. This transition enables repeatable and data-driven decisions that significantly reduce diagnostic uncertainty.

The data used for evaluating belt refurbishment success rates (RSR) and service life were collected and verified by the conveyor belt management team at the Bełchatów lignite mine. The team tracks each belt section’s usage and monitors refurbishment outcomes using internal protocols provided by the regeneration plant. These protocols include detailed reasons for unsuccessful refurbishments. The mine utilizes a belt management software system (“Taśma”), implemented in the mid-1980s, which stores historical data on the installation, dismantling, and operating time of each belt segment. Prior to adopting DiagBelt+, refurbishment decisions were based on belt age and visual inspections. The introduction of in situ scanning has replaced subjective assessments with objective, measurable diagnostics that significantly improve decision quality and traceability.

The DiagBelt+ system was developed and refined as part of an industrial–academic project focused on modernizing belt maintenance strategies. The Bełchatów lignite mine (KWB Bełchatów), Poland’s largest open-pit operation, played a key role in testing and implementing the solution. The mine adopted the system for full-scale deployment in 2023, integrating it into its belt condition monitoring and replacement decision process.

The DiagBelt+ system was launched in mid-2023 and has since been used to analyze approximately 106.5 km of conveyor belts (widths B1800 and B2250, ST 3150 strength class) across more than 20 excavator–conveyor–stacker systems at the Bełchatów lignite mine. Based on the diagnostic results, 62% of the belts were deemed fit for continued operation, 22% were directed for refurbishment, and 16% were scrapped. Diagnostic data enabled optimized decisions, leading to extended service life for 25.5 km of belts and early replacement of 6.1 km due to advanced wear (as of July 2024). From August 2024 to February 2025, an additional 36.9 km of belts across 15 conveyors were scanned. As a result, predicted service life was extended for 21.8 km of belts and shortened for 3.96 km. These decisions reduced the amount of belt waste and significantly cut costs related to new belt purchases, directly improving operational efficiency and ecological performance. The DiagBelt+ system was developed as part of a collaborative industrial–academic project aimed at modernizing conveyor belt maintenance strategies through sensor-based diagnostics. The Bełchatów lignite mine, the largest open-pit operation in Poland, was an active partner in this initiative. In 2023, the mine fully implemented the DiagBelt+ system into its operational belt management process. This real-world deployment played a key role in validating the method’s effectiveness and integrating it into decision-making protocols related to refurbishment, reuse, or disposal of steel-cord belts.

These results confirm the practical advantage of the proposed diagnostic method: compared to earlier decisions based on visual inspection, DiagBelt+ increased the refurbishment success rate from 70% to over 90%, and enabled informed decisions that extended the operation time of 25.5 km of belts and avoided premature disposal.

The DiagBelt+ system underwent extensive validation prior to industrial implementation, including laboratory tests and field trials. The magnetic diagnostic approach it uses is based on technology that has been in use internationally since the late 1970s, and was refined to achieve high resolution imaging—typically one sensor per 1–2 steel cords.

**Figure 12:**
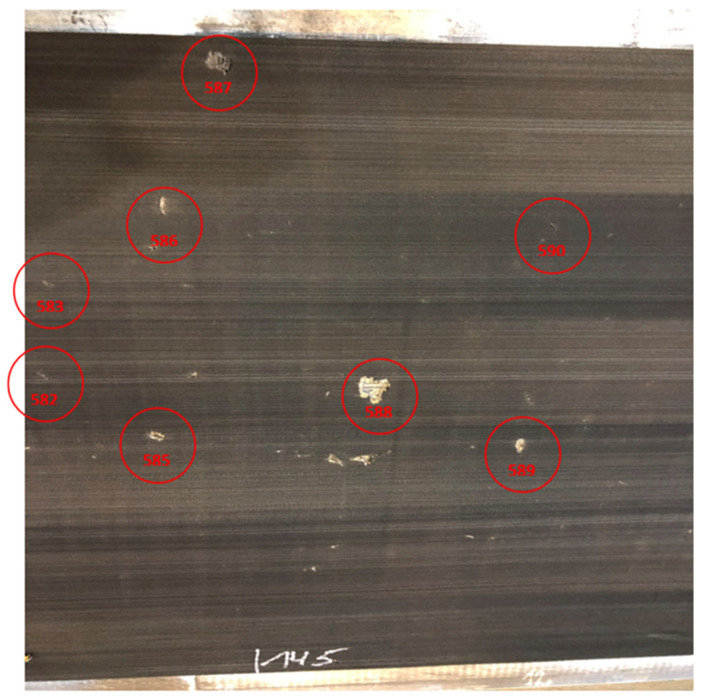
Belt photo after milling of covers.

**Figure 13:**
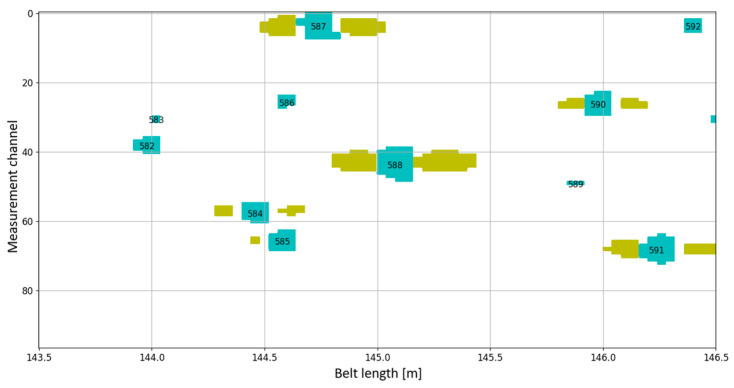
Corresponding magnetic signal. Cyan indicates a signal value of +1, and yellow indicates a value of –1 (associated signal).

As part of the implementation of the NCBR POIR.01.01.01-00-1194/19-00 project, a series of belts operating in a brown coal mine in Poland were examined. This process served as a ground truth validation for the DiagBelt+ system, confirming that the magnetic signals correspond precisely to actual core defects observed during physical inspection. The belts, directed for refurbishment during this project, were first scanned using the DiagBelt+ diagnostic system, and then redirected to the refurbishment process. On the refurbishment station, milling of the supporting and running cover of the belt was carried out (2 mm above the breaker). Visible failures on the belt were located in the form of a signal. Figure 12 and Figure 13 show a fragment of the belt where the occurrence of damage is visible on the remaining, unmilled cover, as well as in the read measurement signal. Failures in Figure 12 are marked with numbering according to the DiagBelt+ system (Figure 13). It should be noted that not all failures visible on the remaining cover are core defects, and these were not marked or registered in the magnetic signal. This confirmed that the system reliably detects internal steel cord damage and distinguishes it from surface-level imperfections not relevant to refurbishment quality.

With a relatively shallow milling of the carrying cover (with preserving the breaker), only very large failures were visible on the milled surface of the belt. On the most damaged sections of both belts, they were identified. The DiagBelt+ system identified a greater number of failures, but some of them may have remained undiscovered during milling when they were not related to piercing the cover to the core with the links. This could include cracked wires and crushed links.

### 4.5. Decision Algorithm and Practical Implementation

**Figure 14:**
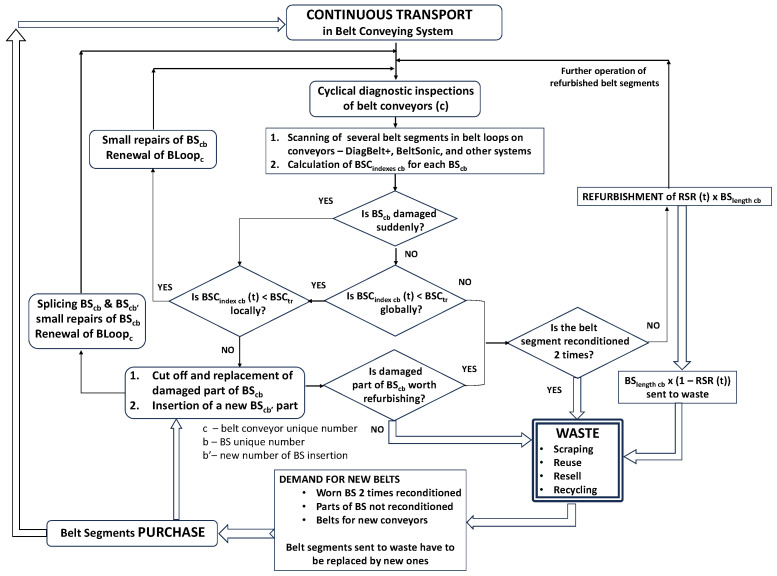
A flow chart of condition-based preventive belt replacement strategy for belt reconditions with the calculation of $BSC_{index}$ for each belt segment (BS) in a belt loop (BL) on each belt conveyor (BC) in the belt conveying system (BCS).

Figure 14 depicts the flowchart of the belt replacement strategy utilizing a diagnostic system.

The decision-making algorithm illustrated in Figure 14 has been applied in practice to support real-time maintenance decisions based on sensor data. Below are several representative cases from the Bełchatów lignite mine demonstrating how the system influenced refurbishment planning:

- Case 1—Belt life extended beyond visual estimate.
  A 2250 mm-wide ST3150 belt with an initial BSCindex of 0.95 defects/m was visually classified for replacement. However, DiagBelt+ scans showed uniform core condition and low deterioration dynamics. The algorithm projected that the threshold value of 1.5 defects/m would not be exceeded for another 8000 operating hours. As a result, the refurbishment was postponed, and the belt remained in use, reducing unnecessary downtime and saving over 1000 m of new belt material.
- Case 2—Belt removed earlier than expected.
  Another segment, 1800 mm-wide with BSCindex at 1.4 defects/m, showed rapid deterioration (slope > 0.1 defects/m per 1000 h). Though visually acceptable, the algorithm forecasted the index would exceed 1.7 within weeks. The belt was preemptively removed and sent for refurbishment, avoiding sudden failure and unplanned stoppage of the KTZ system.
- Case 3—Refurbishment confirmed on schedule.
  A 2250 mm belt planned for refurbishment after 12,000 h of operation had a BSCindex of 1.45 with moderate degradation rate. DiagBelt+ data confirmed that the predicted moment of crossing the 1.5 threshold aligned with the scheduled plan. The refurbishment proceeded as originally planned, validating the previous decision and demonstrating how sensor data can also confirm traditional schedules when appropriate.

The main difference between condition-based replacement using visual inspection and inspections with diagnostic tools is that the subjective and incomplete (based on the external view) assessment of the belt condition is replaced by the reliable assessment of the belt core condition by the magnetic system. Calendar time (the degree of filling the belt service life), which is sensitive to many factors, including a change in the conveyor load, is replaced by an objective measure of the belt condition $B{SC}_{index}$ (e.g., the density of damage per 1 m of belt segment). After crossing the threshold by measured $B{SC}_{index}$ the belt segment will be sent to the refurbishment plant. The threshold ($BSC_{tr}$), identical to all conveyors (regardless of their operating condition, length, belt speed, load, etc.), will be optimally selected, taking into account the changes in the recondition success rate ($RSR\left(t\right)$), costs of refurbishment and belt segment durability after reconditioning. $RSR\left(t\right)$ is not known as a priori but can be calculated as a posteriori after sending several belt segments to refurbishment with known $B{SC}_{index}$ and attained RSR during refurbishment.

For 50 years, a preventive belt replacement strategy to refurbishment was used in the Bełchatów mine based on the belt condition. The belt condition was determined during the visual inspection taking into account their age in comparison to selected service life for a given conveyors (expected time of their operation) working in specific conditions. The use of a subjective visual assessment and operating time, which is a very inaccurate measure of belt condition, led to frequent recondition failures. The belts were pulled off the conveyors too late, because, although the belt did not look worn from the outside, the core condition was poor. Numerous cables and corrosion intersections, invisible from the outside, prevented successful reconditioning. The RSR indicator was about 70%. The solution was to implement magnetic diagnostics of the core condition using the Diagbelt+ system. Determining the core condition became possible on the conveyor, which not only reduced the costs, but above all, enabled an objective assessment of the invisible core and better determination of the moment of dismantling. Currently, the mine is in the phase of intensive scanning of belts directed for refurbishment so that an optimal selection of BSIT will be possible to minimize the costs of using belts in the mine. Savings generated by avoiding dismantling of belts too early turned out to be a big positive surprise. Many belts working on less loaded conveyors have a little used belt core, although they have long filled the temporary Resurs (expected operational time). They would normally be directed to refurbishment, and it would probably be successful. However, greater savings allow further use of the belt for the coming months and years, because the belt loop can continue working without incurring costs of refurbishment. The success of refurbishment depends more on the core condition and less on the condition of the covers and the edges. The use of magnetic diagnostics NDT core condition makes this task easier. This will increase the refurbishment success rate (RSR) up to 90%, extend belt service life, reduce the need for new belt purchases, and improve cost-efficiency. The cost of reconditioning at Bestgum plants is estimated at 50% of the purchase price of the new belt. Leading belt manufacturers are estimated to make up 60–65% of purchase costs.

## 5. Discussion and Conclusions

The work aims to demonstrate the profitability of preventive replacements for belt regeneration. Lignite mines in Poland have successfully employed this strategy for over 50 years. The current market size exceeds $4 billion and is growing at a rate of 3 to almost 6% annually. Various estimates indicate that the production and delivery of belts consume significant resources, with approximately 75% of production costs attributed to raw materials. The remaining costs include energy, which is subject to price increases, and labor.

Many cheaper components are not approved for use in Europe but can be used outside the EU. The pandemic has highlighted extended supply chain timelines and geographical reach (due to carbon leakage), increasing the risk of deadline and quality failures and leading to price hikes, sometimes doubling. Refurbishment has once again become an attractive method to meet growing demand and enhance the security of supply and transport system continuity.

In Poland, belts are refurbished on-site, a practice that originated due to the lack of foreign exchange for imports and continues today due to extended delivery times and related uncertainties. Heavy-weight belts, being the most expensive, undergo refurbishment to maximize their utility. It is considered environmentally wasteful to discard them after a single use. Therefore, in Poland, heavy-weight belts are reconditioned, often twice (with the core being used three times), and in the past, even three times. Textile belts were also reconditioned in the past [51], a practice now rare due to the lower price of belts and the less valuable and durable core. Water ingress into the core causes delamination of the interlayers, excluding the belt from refurbishment.

Correct refurbishment is possible when the core is not excessively damaged. The use of core diagnostics and assessment of its condition on conveyors significantly reduces errors in decision-making during refurbishment. With the implementation of diagnostics using the DiagBelt+ system and the ability to test belt abrasion with the BeltSonic system, the probability of errors decreases, leading to an improved refurbishment success rate (RSR) and increased economic efficiency in the reconditioning process.

In the 1980s, the proportions of new and remanufactured belts on conveyors were 60% new and 40% remanufactured. Current simulations suggest a reversal of these proportions, with 60% remanufactured and 40% new. The Bełchatów mine, following the closure of one of its workings, practically abandoned the purchase of new belts. Instead, it effectively assesses the condition of dismantled belts and makes informed decisions on whether to reuse, refurbish, or discard them. To ensure the environmental effectiveness of the full belt use cycle, efforts are made to resell used belts, use them for other purposes (reuse), or recycle them to recover all components [52,53]. The refurbishment plant already recovers milled rubber from the reconditioning process to produce mats, carpets, and paving stones for parking lots. As used belts become more available, recycling rates will likely need to increase. Rubber from conveyor belts can be recycled in a similar way to rubber from tires [54].

The use of both measurement systems, DiagBelt+ for assessing the technical condition of the core and BeltSonic for evaluating the cover thickness, provides a comprehensive understanding of the technical state of the examined conveyor belt. This approach allows for the continuous monitoring of changes in its condition over time through periodic measurements. This enables the implementation of predictive belt replacement, based on the diagnosed condition, following any preventive replacement strategy. This strategy allows for choosing (for refurbishment only) belts that actually show a significant number of failures but still qualify for refurbishment. Utilizing the refurbishment capabilities of dismantled belt fragments that have reached a level of wear qualifying them for refurbishment increases the working time of full sections, minimizing the risk of emergency replacement and production losses associated with downtime and waiting for the delivery of new belts.

Traditional methods of assessing the condition of belts, such as visual inspection combined with the calendar age of the belt’s operation, proved to be ineffective from the mine’s perspective. As a result of the project implementation and the use of the system, the mine reports significant benefits, avoiding the need to send belts for refurbishment that, despite exceeding the calendar age of operation, still maintain a good technical condition.

The core contribution of this study is demonstrating that quantified, sensor-based diagnostics enable significantly more accurate refurbishment decisions, supporting both cost optimization and sustainability in belt lifecycle management.

## Data Availability

The data presented in this study are available on request from the corresponding author.
